# Antiviral Activity of Sulfated Polysaccharide of *Adenanthera pavonina* against Poliovirus in HEp-2 Cells

**DOI:** 10.1155/2014/712634

**Published:** 2014-08-20

**Authors:** Ananda Marques de Godoi, Lígia Carla Faccin-Galhardi, Nayara Lopes, Daniele Zendrini Rechenchoski, Raimundo Rafael de Almeida, Nágila Maria Pontes Silva Ricardo, Carlos Nozawa, Rosa Elisa Carvalho Linhares

**Affiliations:** ^1^Departamento de Microbiologia, CCB, UEL, 86051-990 Londrina, PR, Brazil; ^2^Departamento de Química Orgânica e Inorgânica, UFC, 60020-181 Fortaleza, CE, Brazil

## Abstract

*Adenanthera pavonina*, popularly known as red-bead tree, carolina, pigeon's eye, and dragon's eye, is a plant traditionally used in Brazil for the treatment of several diseases. The present study aimed at evaluating the activity of sulfated polysaccharide from the *Adenanthera pavonina* (SPLS*Ap*) seeds against poliovirus type 1 (PV-1) in HEp-2 cell cultures. The SPLS*Ap* presented a cytotoxic concentration (CC_50_) of 500 *μ*g/mL in HEp-2 cell cultures, evaluated by the dimethylthiazolyl-diphenyltetrazolium bromide method (MTT). The SPLS*Ap* exhibited a significant antiviral activity, with a 50% inhibitory concentration (IC_50_) of 1.18 *µ*g/mL, determined by plaque reduction assay and a high selectivity index (SI) of 423. The maximum inhibition (100%) of PV replication was found when the SPLS*Ap* treatment was concomitant with viral infection (time 0 h), at all tested concentrations. The maximal inhibition was also found when the SPLS*Ap* was used 1 h and 2 h postinfection, albeit at 50 *μ*g/mL and 100 *μ*g/mL. Therefore, we demonstrated that the SPLS*Ap* inhibited PV growth. We also suggested that SPLS*Ap* inhibited PV in more than one step of the replication, as the mechanism of antiviral action. We, therefore, selected the compound as a potential candidate for further development towards the control of the infection.

## 1. Introduction

For many decades, natural products have been regarded as potential antivirals in parallel to their counterpart antibacterial action, mainly on the ground of empirical practice of healing. The reason for the search for these substances has been encouraged, amongst them, for their low toxicity and the possibility of multistep mechanism of action. The latter effect could represent lesser selective pressure for the emergence of resistant virus strains in comparison to single-targeted drugs. Screening of medicinal plants has shown a vast number of phytochemicals, such as alkaloids, anthraquinones, coumarins, flavonoids, polyphenols, tannins, and terpenoids, among others, active against several viruses [1]. In addition, high-molecular-weight compounds, for instance, sulfated polysaccharides from plants, marine algae, cyanobacteria, and animal sources, have been extensively investigated for this purpose [2]. Studies showed the low cytotoxicity and safety of sulfated polysaccharides [2, 3] and their health beneficial effects. Sulfated polysaccharides from marine seaweeds, for example, among several therapeutic applications, are known to inhibit free radical generation [3]. Further advantages of the use of native or sulfated polysaccharides for medicinal purposes are low production costs, safety, wide acceptability, and their biochemical versatility. Brazil has a great potential for research in this area due to its outstanding biodiversity and ethnopharmacological knowledge of many medicinal plants [4].* Adenanthera pavonina* (Leguminosae) is a plant popularly known as red-bead tree, carolina, pigeon's eye, and dragon's eye [5], common in Brazil and used for reforestation and handicraft and in popular medicine [6, 7]. The seeds, rich in fat and protein, are used as food by Indian tribes [5, 8]. It is used against fever, vomiting, diarrhea, stomach problems, gout, rheumatism, boils, hypertension, pulmonary infections, and chronic ophthalmia, empirically. [8, 9]. Pharmacological effects, such as, antioxidant, anti-inflammatory, analgesic, antihypertensive, anthelmintic, antibacterial, and antifungal effects, have been scientifically demonstrated [10, 11]. Poliovirus (PV) is the etiologic agent of poliomyelitis, a small, nonenveloped positive-stranded RNA virus (genus* Enterovirus*,* Picornaviridae* family) [12]. Currently, poliomyelitis is under control in most parts of the world, but, despite intensive efforts to eradicate the virus, the disease remains endemic in some countries in Africa and Asia, with 407 cases reported in 2013 [13]. The disease has a great epidemiological importance and there is an increasing concern of the world health authorities for the permanent risk it still represents, despite the vaccination program. Moreover, PV is one of the best-understood models of virus and much used in tests for new antiviral drugs. Therefore, we choose PV to evaluate the antiviral effect of the SPLS*Ap *in HEp-2 cell cultures.

## 2. Materials and Methods

### 2.1. Compound: * Adenanthera pavonina (L.)*

Seeds were collected at the Campus do Pici, Universidade Federal do Ceará, Fortaleza, CE, Brazil, in November and December 2010.* A. pavonina* polysaccharide was isolated from the endosperms and extracted with hot water (85°C) [14]. To obtain the sulfated polysaccharide (SPLS*Ap*), 300 mg of galactomannan was allowed to swell in pyridine:N,N-dimethylformamide (50 : 10 v/v) with stirring at 25°C (12 h) until finely dispersed suspensions were obtained. The suspension was cooled to 4°C and 12 mL chlorosulfonic acid was slowly added with stirring over 24 h at 4°C. The resulting solutions were neutralized with saturated aqueous NaHCO_3_, dialyzed (molecular weight cut off 8–12 kDa) for 120 h against distilled water, and the sulfated derivative was collected after lyophilization [15, 16]. The degree of sulfation (DS) was ascertained from the sulfur content (%S) determined by elemental analysis using a Perkin-Elmer CHNS 2400 analyzer and the calculation provided by equation - DS = (1.62 × S%)/(32 − 1.02 × S%) [17].* SPLSAp Fourier transform-IR spectrum was* recorded with a Shimadzu IR spectrophotometer (8300) in the range of 400 and 4000 cm^−1^ as a KBr pellet. The molar mass was determined by gel permeation chromatography with a Shimadzu LC-10AD chromatograph with a RID-10A refractive index detector at 40°C. The analysis was performed with an Ultrahydrogel linear column (7.8 mm × 300 mm), flow rate of 0.5 mL/min, and polysaccharide concentration of 0.1% (w/v), dissolved in water, and 0.1 mol/L sodium nitrate was used as eluent. The intrinsic viscosity [*η*] was determined in a Cannon-Fenske viscometer (Schott-Geräte, AVS-350) with thermostated capillary Schott 520 13 at 25°C ±0.01.

### 2.2. Cells and Virus

HEp-2 cells (epithelial human larynx carcinoma cells, ATCC CCL-23) were grown at 37°C in Dulbecco's modified Eagle's medium (DMEM), supplemented with 10% fetal bovine serum (∗Life Technology Corp., USA), and treated with 100 *μ*g/mL streptomycin (∗), 100 IU/mL of penicillin (Novafarma Indústria Farmacêutica, BR), and 2.5 *μ*g/mL of amphotericin B (Meizler Biopharma, BR). Poliovirus type-1 (PV-1), ATCC, VR-58, was propagated in HEp-2 cell cultures and stored at −20°C with 10% glycerol. The virus titer was determined by plaque assay.

### 2.3. Cytotoxicity Assay

The SPLS*Ap* cytotoxicity was determined by MTT kit assay (Sigma Chem. Co., USA), according to the manufacturer's instructions. Briefly, 70% confluent HEp-2 cell cultures grown in 96-well microplates (Nunc A/S, Denmark) were treated with varying concentrations of the SPLS*Ap *(50 *μ*g/mL to 1000 *μ*g/mL) and maintained at 37°C with 5% CO_2_ for 72 h. The overlay medium was replaced with 10 *μ*L of the MTT reagent (1.25 *μ*g/mL) and incubated as before, for 3 h. This was followed by the addition of 90 *μ*L of the solubilizer agent and after 15 min the absorbance read at 570 and 690 nm. The percentage of cell viability (%CV) was calculated by the formula %CV = [100 − (At/Ac) × 100], where At and Ac refer to the absorbance of test substance and control (untreated cells), respectively [18]. The concentration of the SPLS*Ap* capable of reducing cell viability by 50% compared to cell control and calculated by linear regression analysis corresponds to the 50% cytotoxic concentration (CC_50_).

### 2.4. Plaque Reduction Assay

The antiviral activity of the SPLS*Ap* was determined by plaque reduction assay (PRA) according to Lopes et al. [19]. HEp-2 cells grown in 24-well plates (TPP, CH) to 100% confluence were infected with PV (50 to 100 PFU) and treated simultaneously with varying concentrations of the SPLS*Ap* (0.78 to 100 *μ*g/mL). These cell cultures were overlaid with nutrient agarose (DMEM 2x/1.8% agarose [v/v]) containing 25 mM MgCl_2_. After 40-hour incubation, the cells were fixed with 10% formaldehyde in phosphate-buffered saline (PBS), pH 7.3, for 24 hours. Cells were stained with 0.5% crystal violet in 20% ethanol. The plaques were counted and percentage of viral inhibition (%IV) was calculated as [1 − (*V*_*d*_/*V*_*c*_)] × 100, where *V*_*d*_ and *V*_*c*_ refer to the number of plaques in the presence and absence of the compound, respectively [20]. The concentration of the SPLS*Ap* that inhibited 50% PFU (IC_50_) was calculated by linear regression analysis and the selectivity index (SI) was expressed by the ratio CC_50_/IC_50_. Human alfa-2B interferon (Meizler Biopharma S/A, BR) was used as positive control.

#### 2.4.1. Time-of-Addition Assay

It was performed according to Yamamoto et al. [18] in that varying concentrations of the SPLS*Ap* (12.5 *μ*g/mL, 25 *μ*g/mL, 50 *μ*g/mL, 100 *μ*g/mL) were added to the cell cultures simultaneously (0 h) and before (−1 and −2 h) and after (1, 2, 4, and 8 h) the infection, followed by PRA.

#### 2.4.2. Virucidal Effect

A virus suspension was incubated with varying concentrations of the SPLS*Ap* (12.5–100 *μ*g/mL) (v/v) for 1 h at 37°C in water bath, diluted to the tenth and the residual infectivity determined by PRA [21].

#### 2.4.3. Inhibition of Adsorption Assay

The cell monolayers previously maintained at 4°C for 1 hour were infected in the presence of the SPLS*Ap*, at the same concentrations, as before, and kept for further 80 min at 4°C. The cell cultures were washed with cold PBS to remove nonadsorbed virus followed by PRA [22].

### 2.5. Immunofluorescence Assay (IFA)

HEp-2 cell cultures grown on coverslips, in 24-well plates, were infected with 500 *μ*L of PV and simultaneously treated with varying concentrations of the SPLS*Ap* (12.5 *μ*g/mL, 25 *μ*g/mL, 50 *μ*g/mL, and 100 *μ*g/mL). Cultures of infected and untreated cells and cultures of untreated and noninfected cells were maintained for controls. After 24 h, the cells were washed with 0.05% Tween 20 PBS, fixed with cold acetone (−20°C), and blocked with 2% powdered skim milk PBS. The cells were incubated for 30 min at 37°C with rabbit anti-PV-1 serum (INCQS-Fiocruz, BR), further washed three times with Tween 20 PBS, and incubated for 30 min at 37°C, with sheep anti-rabbit IgG FITC conjugate (Sigma Chem. Co., EUA) [23]. The cells were examined in a Zeiss fluorescence microscope (Zeiss Axio Imager A1). A total of 100 cells/coverslip were randomly chosen and the percentage of fluorescent cells was calculated in comparison to nonfluorescent cells.

### 2.6. RT-PCR

RNA of cells infected and treated with SPLS*Ap *at 1.56 *μ*g/mL to 25 *μ*g/mL and that of the respective controls (human alpha-2B interferon, virus, and cell controls) were extracted with QIAamp RNA Mini Kit. The first reaction mixture consisting of 5 pmol of random primer, 1 mM dNTP, and RNAse-free water up to 7 mL was prepared. Seven microliters of the first reaction mixture and 5 *μ*L of the extracted viral RNA were incubated for 5 min at 65°C. A second reaction mixture consisting of 1x M-MLV reaction buffer, 0.01 M DTT, 100 U M-MLV (Moloney Murine Leukemia Virus) reverse transcriptase, and RNAse-free water up to 8 *μ*L was prepared. Eight microliters of second reaction mixture was added to each sample and sequentially incubated for 10 min at 25°C, 50 min at 37°C, and 15 min at 70°C. The PCR reaction was carried out at a final volume of 25 *μ*L containing 0.4 mM dNTP, 2 pmol of each primer, 2.5 U Taq DNA polymerase with PCR buffer, and cDNA. Specific primers for PV capsid gene (VP1-VP4) were 5′AGTTTCACCGAAGGCGGA 3′(F) and 5′CGCTGACACAAAACCAAGGA 3′(R), resulting in a 102 bp amplified product. The PCR program consisted of denaturation at 95°C for 10 min, followed by 40 cycles by denaturation for 15 sec at 95°C, annealing at 60°C for 1 min, and extension for 1 min at 72°C. The final cycle of extension was 7 min at 72°C. Ten-microliter aliquots of the PCR products were resolved in 12% polyacrylamide gel electrophoresis [19].

### 2.7. Statistical Analysis

Anova followed by Tukey's test (BioEstat 5.0 for Windows XP, 2007) was used to determine the difference among the SPLS*Ap* experiments and control groups. Values of *P* < 0.05 were considered significant.

## 3. Results

### 3.1. Characterization of the SPLS*Ap*

The presence of sulfated groups was identified by infrared spectrum and presented 13.6% of amount of S (S%) and degree of sulfation (DS) 1.21. The compound presented an absorption at 1257 cm^−1^, assigned to the asymmetric stretching vibration of the S=O linkage [24, 25] and showed characteristic absorption bands of the galactomannans, as reported elsewhere [26–30]. The *η* for SPLS*Ap* was 3.2 dL/g and the molar mass was 7.0 × 10^5^ g/mol similar to those reported for sulfated galactomannans for* M. scabrella* (6.20 × 10^5^ g mol^−1^) [31] and* L. leucocephala* (5.74 × 10^5^ g mol^−1^) [16].

### 3.2. Anti-PV Activity of the SPLS*Ap*

**Figure 1:**
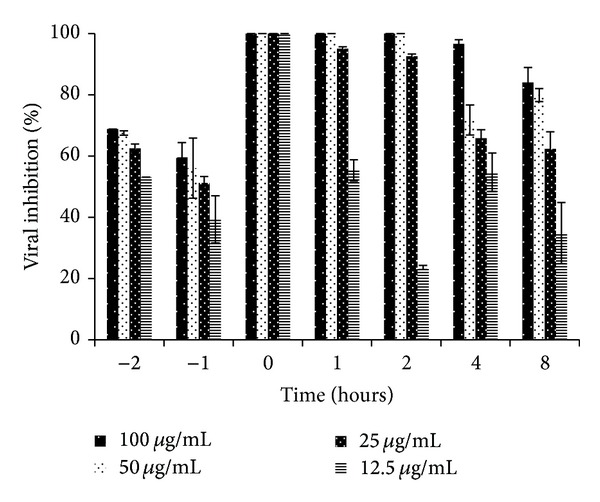
Effect of the time-of-addition assay of the* Adenanthera pavonina *sulfated polysaccharide (SPLS*Ap*) on poliovirus type 1 (PV-1) in HEp-2 cells by plaque reduction assay. The percentage of viral inhibition (%VI) is expressed as mean ± SD of triplicate independent experiments.

**Figure 2:**
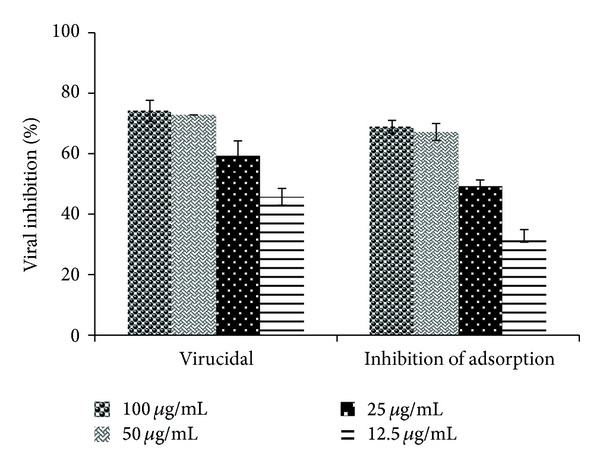
The effect of the* Adenanthera pavonina* sulfated polysaccharide (SPLS*Ap*) on poliovirus type 1 (PV-1) particle (virucidal activity) and in virus adsorption on HEp-2 cells by plaque reduction assay. The percentage of viral inhibition (%VI) is expressed as the mean ± SD of triplicate independent experiments.

**Figure 3:**
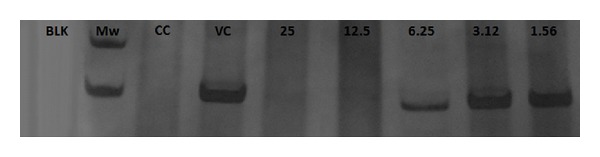
The effect of the* Adenanthera pavonina* sulfated polysaccharide (SPLS*Ap*) in the synthesis of PV-1 RNA monitored by RT-PCR. Polyacrylamide gel electrophoresis of amplicon for PV-1* VP1-VP4* (102 bp). Negative control (BLK); MW marker (Mw); cell control (CC); virus control (VC), and PV-1 treated with SPLS*Ap *at the indicated concentrations (1.56–25 *μ*g/mL).

Table 1 shows theSPLS*Ap* CC_50_ (500 *μ*g/mL) and its antiviral activity under the assays of the time of addition (0 h), virucidal and inhibition of adsorption, and the respective SI. The most efficient effect is indicated at the time 0 h by the IC_50_ 1.18 *μ*g/mL and the SI 423.73.  Figure 1 shows that the maximum inhibitory effect was found at this time period (time 0 h), at all tested concentrations. Times 1 and 2 h postinfection (pi.) also demonstrated high %VI, 95% and 93%, respectively, at 25 *μ*g/mL, and 100% at 50 and 100 *μ*g/mL, at both times. When the SPLS*Ap* was added at 4 and 8 h pi., a slight reduction was observed; however, the inhibition remained above 70% for the two highest concentrations. Lower effect was detected at 1 and 2 h before infection with inhibition ranging from 39% to 69%, for all tested concentrations.  Figure 2 shows the direct effect of SPLS*Ap* on PV-1 (virucidal activity) resulting in inhibition of 74.1%, 72.8%, 59.3%, and 45.7% at 100 *μ*g/mL, 50 *μ*g/mL, 25 *μ*g/mL, and 12.5 *μ*g/mL, respectively. The inhibition of adsorption assay presented the following figures 68.9%, 67.2%, 49.2%, and 32.8%, respectively, for the same concentrations. Interferon, used as positive control in PRA, inhibited 100% of PV-1 at the concentration of 10,000 U/mL. The IFA was carried out to evaluate the effect of the SPLS*Ap* in the expression of viral protein. It was performed with cells treated at the time 0 h of infection, at the concentrations of 12.5 *μ*g/mL to 100 *μ*g/mL and the inhibition of fluorescent cells varied from 70.9% to 100% (Table 2). The inhibition of viral RNA by the compound was demonstrated by RT-PCR. The SPLS*Ap* inhibited viral RNA synthesis at the concentrations 12.5–100 *μ*g/mL. The replicon was visualized only at low concentrations of the SPLS*Ap*—6.25 *μ*g/mL, 3.12 *μ*g/mL, and 1.56 *μ*g/mL (Figure 3). The replicon of cells infected and treated with human alfa-2B was not observed in the electrophoresis gel, and therefore, not shown.

## 4. Discussion

Currently, the anti-PV activity of natural products has been stimulated. Soltan and Zaki [32] reported that four out of forty-two Egyptian medicinal plants showed antiviral effect against PV. The anti-PV activity was also demonstrated with extract from* Avicennia marina* [33], with an* Agaricus brasiliensis* polysaccharide [22] and with four polysaccharides from* Azadirachta indica* [23]. In this study, we demonstrated the anti-PV action of the SPLS*Ap*. The SPLS*Ap* showed low cytotoxicity and IC_50_ (1.18 *μ*g/mL) resulting in a high SI, one of the features sought for a potential candidate for antiviral. Similarly, IC_50_ (1.73 *μ*g/mL) was found with a sulfated polysaccharide from* Caesalpinia ferrea* seeds, also a galactomannan [18]. According to Cerqueira et al. [26], the galactomannans extracted from seeds of numerous plants (particularly theLeguminosae) are polysaccharides built up of a *β*-(1-4)-d-mannan backbone with single d-galactose branches linked *β*-(1-6), with predominance of *β*-glucans, to which the antiviral activity may be attributed.

The time-of-addition assay was performed to investigate the possible steps of the SPLS*Ap* action mechanism in the replication of PV. The SPLS*Ap* used at the times 0, 1, 2, 4, and 8 h p.i (Figure 1) showed that the maximum PV inhibition was observed at the time 0 h, suggesting that the compound inhibited mainly the initial stages of viral infection. This result is in agreement with reports on the antiviral property of high-molecular-weight compounds. Sulfated polysaccharides are among the most studied and have demonstrated an* in vitro *broad spectrum of antiviral activity. This property is thought to be related to distinct structural characteristics and not only to high charge density or chain length. These compounds can potentially block early stages of viral replication, including attachment to the target cell and viral entry [2, 34]. The sulfation provides a negative charge density greater than natural compound, interfering most efficiently with electrostatic interactions between the positive charges of the viral proteins and cell receptors, usually with negative charges [2, 35]. To substantiate this feature, we analyzed the SPLS*Ap* effect directly on viral particles (virucidal assay) and its interaction with cell receptors (inhibition of adsorption assay) resulting in low activity. The presence of *β*-glucans in the SPLS*Ap *and the possibility of interferon induction cannot be ruled out, as suggested by Biesert et al. [36]. We also demonstrated that SPLS*Ap* showed effect when used after infection. The SPLS*Ap* could also present low-molecular-weight polysaccharide components eliciting antiviral effect in steps after virus entry into the cells [2]. This hypothesis could explain the inhibition of viral protein expression or the posttranslational polyprotein cleavage and the inhibition of viral RNA synthesis, as we also demonstrated, and concur with the whole SPLS*Ap* anti-PV effect.

## 5. Conclusion

Our results showed that SPLS*Ap *inhibits the growth of PV in more than one step of its replicative cycle, suggesting more than one mechanism of antiviral action. In addition, the compound is endowed with a low cytotoxicity and, therefore, can be a candidate for further development of an antipoliovirus agent.

## Figures and Tables

**Table 1:** The antiviral activity of the *Adenanthera pavonina* sulfated polysaccharide (SPLS*Ap*) against poliovirus. Evaluation under the assays of the time of addition (0 h), virucidal, and inhibition of adsorption, monitored by plaque reduction assay (PRA).

Assay	PV-1		
	CC_50_^a^	IC_50_^b^	SI^c^
Time of addition (0 h)	500	1.18	423.73
Virucidal	500	16.47	30.54
Inhib. of adsorption	500	25.25	19.80

^a^Fifty percent cytotoxic concentration (*µ*g/mL).

^b^Fifty percent inhibitory concentration (*µ*g/mL).

^c^Selective index (CC_50_/IC_50_).

**Table 2:** The effect of the *Adenanthera pavonina* sulfated polysaccharide (SPLS*Ap*) in the synthesis of PV-1 protein by immunofluorescence assay. The SPLS*Ap* was used at the time 0 h of the infection at the indicated concentrations. Results are expressed by the mean ± SD of the fluorescent cells number and the corresponding % viral inhibition (% VI).

SPLS*Ap* (*µ*g/mL)	Fluorescent cells number	% VI
0	79.0 ± 1.4	—
12.5	22.5 ± 2.12	70.9
25	17.5 ± 0.7	82.6
50	9.0 ± 1.4	92.2
100	nill	100

## References

[1] Hupfeld J, Efferth T (2009). Drug resistance of human immunodeficiency virus and overcoming it by natural products. *In Vivo*.

[2] Ghosh T, Chattopadhyay K, Marschall M, Karmakar P, Mandal P, Ray B (2009). Focus on antivirally active sulfated polysaccharides: from structure-activity analysis to clinical evaluation. *Glycobiology*.

[3] Josephine A, Amudha G, Veena CK, Preetha SP, Rajeswari A, Varalakshmi P (2007). Beneficial effects of sulfated polysaccharides from *Sargassum wightii* against mitochondrial alterations induced by Cyclosporine A in rat kidney. *Molecular Nutrition & Food Research*.

[4] Cunha PLR, Paula RCM, Feitosa JPA (2009). Polissacarídeos da biodiversidade brasileira: uma oportunidade de transformar conhecimento em valor econômico. *Química Nova*.

[5] Sultana R, Gulzar T (2012). Proximate analysis of *Adenanthera pavonina* L. seed oil, a source of lignoceric acid grown in Pakistan. *Journal of the American Oil Chemists' Society*.

[6] Fonseca S, Perez S (2003). Ação do polietilenoglicol na germinação de sementes de *Adenanthera pavonina* L. e o uso de poliaminas na atenuação do estresse hídrico sob diferentes temperaturas. *Revista Brasileira de Sementes*.

[7] Soomro RK, Sherazi STH (2012). Spectroscopic and chromatographic evaluation of the wax ester fraction of *Adenanthera pavonina* oil. *Industrial Crops and Products*.

[8] Krishnaveni A, Selvi S, Mohandass S (2011). Antidiabetic, hypolipidemic activity of *Adenanthera pavonina* seeds in alloxan induced diabetic rats. *Journal of Pharmacy Research*.

[9] Adedapo ADA, Osude YO, Adedapo AA (2009). Blood pressure lowering effect of Adenanthera pavonina seed extract on normotensive rats. *Records of Natural Products*.

[10] Ara A, Saleh-e-In MM, Ahmed NU, Ahmed M, Hashem MAH, Bachar SC (2010). Phytochemical screening, analgesic, antimicrobial and anti-oxidant activities of bark extracts of *Adenanthera pavonina* l. (Fabaceae). *Advances in Natural and Applied Sciences*.

[11] Soares JR, de Carvalho AQ, Dos Santos IS (2012). Antimicrobial peptides from *Adenanthera pavonina* L. Seeds: characterization and antifungal activity. *Protein and Peptide Letters*.

[12] Racaniello VR, Knipe DM, Howley PM (2007). Picornaviridae: the viruses and their replication. *Fields Virology*.

[13] Global Polio Eradication Iniciative Annual report 2014.

[14] Vieira ÍGP, Mendes FNP, Gallão MI, de Brito ES (2007). NMR study of galactomannans from the seeds of mesquite tree (*Prosopis juliflora* (Sw) DC). *Food Chemistry*.

[15] O’Neill AN (1995). Sulphated derivatives of laminarin. *Canadian Journal of Chemistry*.

[16] Ono L, Wollinger W, Rocco IM, Coimbra TLM, Gorin PAJ, Sierakowski M (2003). In vitro and in vivo antiviral properties of sulfated galactomannans against yellow fever virus (BeH111 strain) and dengue 1 virus (Hawaii strain). *Antiviral Research*.

[17] Wang L, Li X, Chen Z (2009). Sulfated modification of the polysaccharides obtained from defatted rice bran and their antitumor activities. *International Journal of Biological Macromolecules*.

[18] Yamamoto KA, Galhardi LCF, Rincão VP (2013). Antiherpetic activity of an *Agaricus brasiliensis* polysaccharide, its sulfated derivative and fractions. *International Journal of Biological Macromolecules*.

[19] Lopes N, Faccin-Galhardi LC, Espada SF (2013). Sulfated polysaccharide of Caesalpinia ferrea inhibits herpes simplex virus and poliovirus. *International Journal of Biological Macromolecules*.

[20] Nishimura T, Toku H, Fukuyasu H (1977). Antiviral compounds. XII. Antiviral activity of amidinohydrazones of alkoxyphenyl-substituted carbonyl compounds against influenza virus in eggs and in mice. *Kitazato Archives of Experimental Medicine*.

[21] Minari MC, Rincão VP, Soares SA, Ricardo NMPS, Nozawa C, Linhares REC (2011). Antiviral properties of polysaccharides from *Agaricus brasiliensis* in the replication of bovine herpesvirus 1. *Acta Virologica*.

[22] Faccin LC, Benati F, Rincão VP (2007). Antiviral activity of aqueous and ethanol extracts and of an isolated polysaccharide from *Agaricus brasiliensis* against poliovirus type 1. *Letters in Applied Microbiology*.

[23] Faccin-Galhardi LC, Aimi Yamamoto K, Ray S, Ray B, Carvalho Linhares RE, Nozawa C (2012). The in vitro antiviral property of *Azadirachta indica* polysaccharides for poliovirus. *Journal of Ethnopharmacology*.

[24] Mähner C, Lechner MD, Nordmeier E (2001). Synthesis and characterisation of dextran and pullulan sulphate. *Carbohydrate Research*.

[25] Yang J, Du Y, Wen Y, Li T, Hu L (2003). Sulfation of Chinese lacquer polysaccharides in different solvents. *Carbohydrate Polymers*.

[26] Cerqueira MA, Souza BWS, Simões J (2011). Structural and thermal characterization of galactomannans from non-conventional sources. *Carbohydrate Polymers*.

[27] Hu C, Kong Q, Yang D, Pan Y (2011). Isolation and structural characterization of a novel galactomannan from *Eremurus anisopterus* (Ker. et Kir) Regel roots. *Carbohydrate Polymers*.

[28] Shobha MS, Vishu Kumar AB, Tharanathan RN, Koka R, Gaonkar AK (2005). Modification of guar galactomannan with the aid of *Aspergillus niger* pectinase. *Carbohydrate Polymers*.

[29] Mudgil D, Barak S, Khatkar BS (2012). X-ray diffraction, IR spectroscopy and thermal characterization of partially hydrolyzed guar gum. *International Journal of Biological Macromolecules*.

[30] Figueiró SD, Góes JC, Moreira RA, Sombra ASB (2004). On the physico-chemical and dielectric properties of glutaraldehyde crosslinked galactomannan-collagen films. *Carbohydrate Polymers*.

[31] Chrestani F, Sierakowski MR, de Andrade Uchoa DE (2009). In vitro antiherpetic and antirotaviral activities of a sulfate prepared from *Mimosa scabrella* galactomannan. *International Journal of Biological Macromolecules*.

[32] Soltan MM, Zaki AK (2009). Antiviral screening of forty-two Egyptian medicinal plants. *Journal of Ethnopharmacology*.

[33] Zandi K, Taherzadeh M, Tajbakhsh S, Yaghoubi R, Rastian Z, Sartavi K (2008). Antiviral activity of *Avicennia marina* leaf extract on HSV-1 and vaccine strain of polio virus in vero cells. *International Journal of Infectious Diseases*.

[34] Song X, Yin Z, Li L (2013). Antiviral activity of sulfated *Chuanminshen violaceum* polysaccharide against duck enteritis virus in vitro. *Antiviral Research*.

[35] Saha S, Galhardi LCF, Yamamoto KA (2010). Water-extracted polysaccharides from Azadirachta indica leaves: structural features, chemical modification and anti-bovine herpesvirus type 1 (BoHV-1) activity. *International Journal of Biological Macromolecules*.

[36] Biesert L, Adamski M, Zimmer G (1990). Anti-human immunodeficiency virus (HIV) drug HOE/BAY 946 increases membrane hydrophobicity of human lymphocytes and specifically suppresses HIV-protein synthesis. *Medical Microbiology and Immunology*.

